# A polymeric nitrogen N$_6$–N$_2$ system with enhanced stability at low pressure

**DOI:** 10.1038/s41598-022-19080-0

**Published:** 2022-09-12

**Authors:** El Mostafa Benchafia, Xianqin Wang, Zafar Iqbal, Sufian Abedrabbo

**Affiliations:** 1 Department of Physics, Khalifa University, Abu Dhabi, UAE; 2 Department of Chemical, Biological and Pharmaceutical Engineering, New Jersey Institute of Technology, Newark, NJ 07102 USA; 3 Department of Physics, University of Jordan, Amman, Jordan; 4 Department of Chemistry and Environmental Science, New Jersey Institute of Technology, Newark, NJ 07102-1982 USA

**Keywords:** Energy storage, Chemistry, Physics

## Abstract

Postulated in 1992 and synthesized in 2004 above 2000 K and 110 GPa, the singly-bonded nitrogen cubic gauche crystal (cg-PN) is still considered to be the ultimate high energy density material (HEDM). The search however has continued for a method to synthesize cg-PN at more ambient conditions or find HEDMs which can be synthesized at lower pressure and temperature. Here, using ab initio evolutionary crystal prediction techniques, a simpler nitrogen-based molecular crystal consisting of N$_6$ and N$_2$ molecules is revealed to be a more favorable polynitrogen at lower pressures. The energetic gain of  534 meV/atom over cg-PN and  138 meV/atom over the N$_8$ molecular crystal at zero pressure makes the N$_6$–N$_2$ system more appealing. Dynamical and mechanical stabilities are investigated at 5 and 0 GPa, and vibrational frequencies are assessed for its Raman and IR spectra. The prospects of an experimental synthesis of the N$_6$–N$_2$ polymeric system compared to cg-PN is higher because the C$_{2h}$ symmetry of N$_6$ within this crystal would be easier to target from the readily available N$_3^-$ azides and the observed N$_{3}^+$ and N$_{3}^*$ radicals.

## Introduction

**Figure 1:**
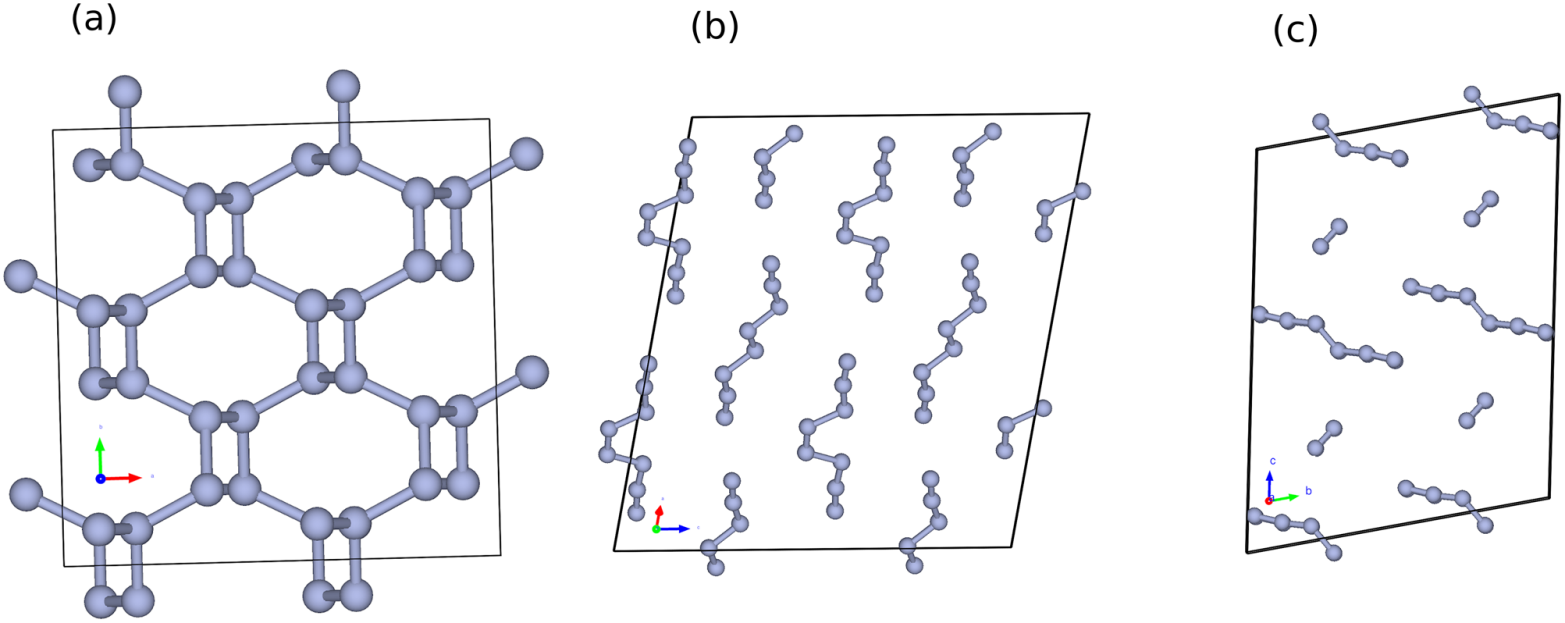
The crystal arrangement of three polymeric nitrogen compounds: (**a**) cg-PN predicted in 1992 and synthesized in 2004 by compressing N$_2$ above 110 GPa and 2000 K. (**b**) The 2N$_8$ molecular crystal predicted in 2013 and synthesized in 2018 by compressing hydrazinium azide above 40 GPa. (**c**) The molecular N$_6$–N$_2$ system investigated computationally in this work at zero pressure.

**Figure 2:**
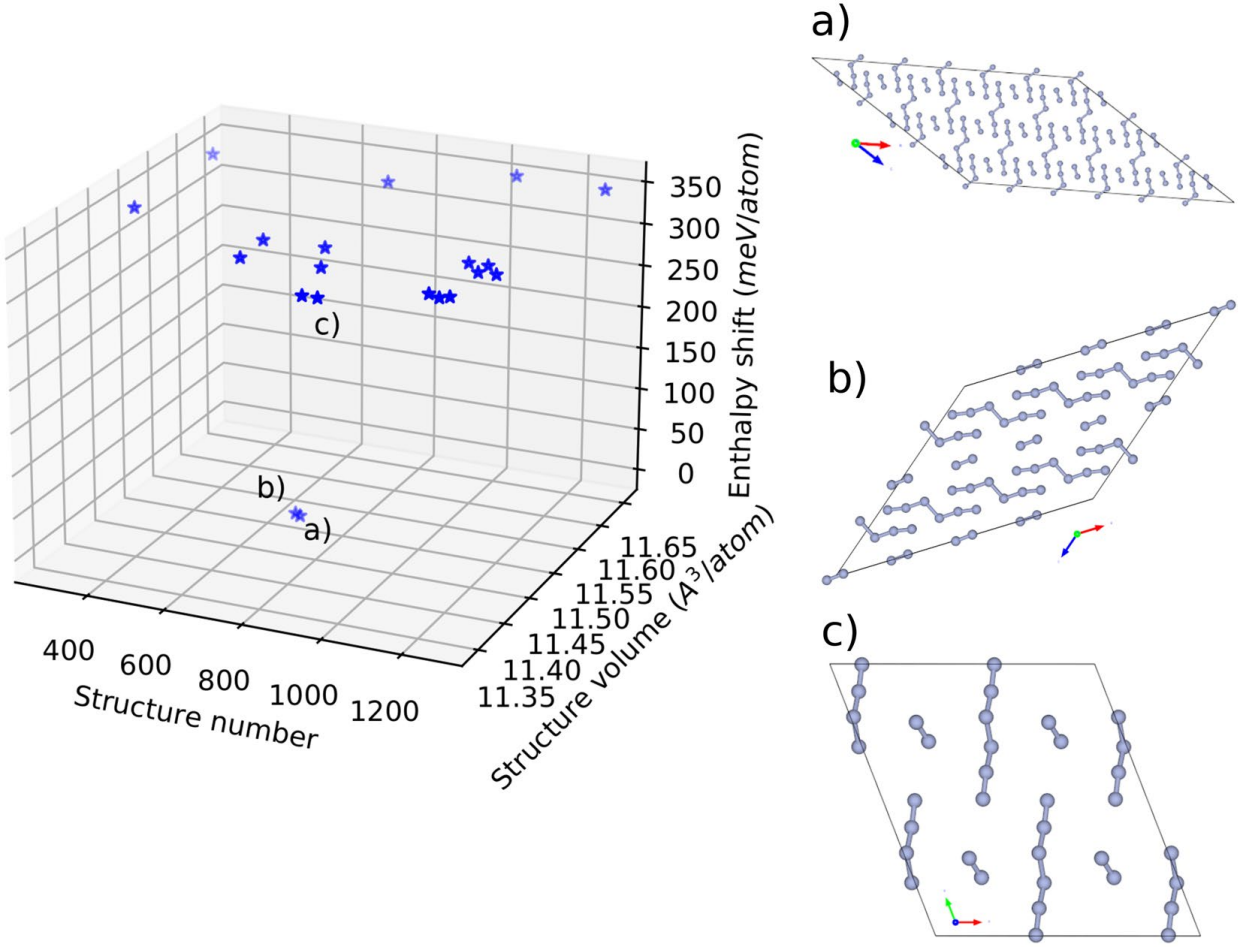
Results of the evolutionary search with USPEX in combination with Quantum Espresso DFT engine for geometry optimization. 41 Generations for a total of 1292 crystal structures were produced for optimization. The best 20 structures were plotted against their structure number along the search, their corresponding volume and enthalpy. Ball and stick representation of the best 3 structures (**a**, **b** and **c**) are presented along with their position in the enthalpy/volume V-H landscape. The enthalpy shift is from the best structure (structure a)) in meV/atom).

With a bond dissociation enthalpy of 225.8 Kcal/mole^1^, molecular nitrogen N$\equiv$N has one of the strongest bonds in chemistry, it is therefore abundant as the major constituent in air and is used as an inert gas in many processes. This exceptional bond strength is only surpassed by another N$\equiv$N bond in the [HNNH]$^{2+}(^1\sum _g^+)$ molecule^2^. As a result, nitrogen compounds are highly stable. Accordingly, any metastable form of nitrogen compounds where nitrogen bonds are single or double, are considered energy storage media. As an example, the car air bag chemistry takes advantage of this unique nitrogen property by using nitrogen in the form of sodium azide NaN$_3$. These azide salts are highly metastable and can be deployed to transform into nitrogen gas upon collision, inflating the airbags and hence saving human lives. The term high energy density materials (HEDMs) is used for compounds that have this capability of storing energy. Because of the above-mentioned properties, the element nitrogen is center stage in almost all explosives, propellants and nuclear warheads as it enters in the fabric of many HEDMs, such as TNT, HMX, and RDX. However, all these compounds often lead to post detonation by-products in the form of nitrogen and carbon oxides with serious health and environmental consequences. An all-nitrogen (often referred to as polynitrogen or polymeric nitrogen and abbreviated PN) compound will thus be considered a green HEDM, by leveraging its potential of only releasing N$_2$. Historically, a transformation from the molecular N$\equiv$N to an extended network of covalently bonded nitrogen atoms started within the high-pressure physics community as early as 1984. In the work of Nellis et al.^3^, this transition was observed at the moderate pressure of 300 kbar (0.03GPa) but at an elevated temperature of 2000 K. The finding triggered a big scientific interest, starting with the computational work of McMahan et al.^4^ in 1985. The finding suggests the sought-after transformation from molecular N$_2$ to a polymeric nitrogen in the form of a simple cubic phase to take place within 1 Mbar (100 GPa). Other polymorphs such as the A7 distorted sc and the black-phosphorous structure were also suggested to potentially be adopted by PN with a far larger volume change upon transition. A countless number of polymeric nitrogen compounds were predicted over the years. They can be classified into three categories: (1) crystals at different ranges of pressure^5–13^, (2) isolated molecules or macromolecules and ions as stand-alone stable entities in their gas phase^14,15^ and (3) nitrogen clusters that require host materials, such as carbon nanotubes or graphene as substrates to enhance their stability^16,17^. Experimental achievements are far less abundant and can be briefly summarized categorically into: (1) he ephemerally observed compounds that were detected like N$_3^+$ and N$_4^+$ encountered in electrical discharges such as plasma^18–20^, (2) he N$_5^+$ cation first synthesized in 1999 in the N$_5$AsF$_6$ salt by Christe et al.^21^ and was followed by many other N$_5^+$ salts with varying sensitivity vis á vis temperature and experimental handling^22–24^, such as the N$_5^-$ anion in 2017 from the pioneering work of Zhang et al.^25^, (3) the high pressure phases such as cg-PN (Fig. 1-a) in 2004 in the work of Eremets et al.^26^ in addition to the synthesis of the molecular crystal N$_8$ (Fig. 2-b) by Duwal et al.^27^, and (4) syntheses of polymeric nitrogen compounds^28–31^ stabilized within the walls of carbon nanotubes, such as N$_8^-$ and cg-PN.

The field of polymeric nitrogen enters in the realm of new materials discovery in the broader sense. Carbon, the nearest neighbor to nitrogen in the periodic table, is a good example of the achievements than can be made in this process. C$_{60}$ synthesis broke many speculations as early as 1985 (interestingly around the same time when polymeric nitrogen was just starting to emerge) and was followed by carbon nanotubes and then graphene. The huge developments made so far for materials discovery are only speeding up this process. For instance, the USPEX^32–34^ algorithm made interesting predictions regarding superconductors^35–38^, magnetic materials^39,40^ and materials that changed many perspectives of conventional chemistry^41,42^. Concerning polymeric nitrogen and HEDMs in general, we can find interesting implementation of this method in the work of Pakhnova et al.^43^ for explosive materials. Of interest is also the predicted polynitrogen in the work of Bondarchuk et al.^44^, consisting of bipentazole N$_{10}$ in the form of two connected cyclic N$_5$ and claimed to possess higher stability than the EZE-N$_8$ molecule^13^. A strategic synthesis of this HEDM has been suggested from N$_5$ cyclic radicals but is yet to be made. The N$_6$–N$_2$ system presented in this report is the result of such implementation of the USPEX^32–34^ algorithm. Instead of starting the search without any constraints which eventfully leads to N$_2$ phases at low pressures, we constrained the search to look for a molecular crystal with N$_6$ and N$_2$ predefined blocks. This resulted in finding a system with higher intrinsic stability than the polynitrogens previously found.

It is important to mention that there are many hurdles facing polymeric nitrogen compounds to form at ambient conditions. A system having a local minimum in the potential energy surface (PES) instead of a global minimum can have a short lifetime or stay elusive for detection in experimental procedures. Diamond for instance is a classical example of a system lacking the thermodynamic stability in comparison to graphite. However, diamond does not reverse back to graphite once it is formed as the energy barrier to reverse to graphite is too high. Moreover, the high pressure and temperature (HPHT) required to synthesize artificial diamond were reduced tremendously over the years by implementing plasma chemical vapor deposition (CVD). For instance, the temperature and pressure requirements for diamond synthesis are as high as (30120 GPa, 1000–3000K) with shock waves, (15 GPa, 3000 -3500K) with HPHT, (5–10 GPa, 1500–2000K) with catalytic HPHT and (<1 GPa, <1500K) with plasma CVD^45^. The experimental work conducted in our group to synthesize the cubic gauche by plasma CVD is an example of the efforts within the scientific community to synthesize polymeric nitrogen compounds despite their metastability at ambient conditions^31,46^. Another key factor for a potential synthesis of a polynitrogen is a high energy lattice structure which has the capability of acquiring enough cohesive energy to overcome metastability. Also, the lower the pressure regime for these compounds to exist, the higher their likelihood to be quenched at ambient conditions. The N$_6$–N$_2$ system we suggest in this contribution is thus believed to satisfy many requirements towards a synthesis.

## Results and discussion

**Figure 3:**
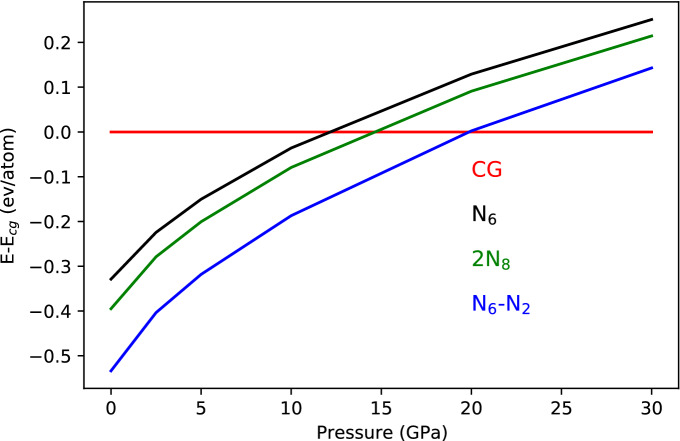
Enthalpy shift from cg-PN of the molecular crystals N$_6$–N$_2$, the 2N$_8$ from the work of Hirschberg et al.^13^ and the N$_6$ solid from Greschner et al.^57^. The N$_6$–N$_2$ system is energetically more favorable than 2N$_8$in the whole pressure range from 0 to 30 GPa. Meanwhile, cg-PN becomes more favorable only after 20 GPa.

The USPEX^32–34^ algorithm and the DFT^47,48^ Quantum Espresso^49,50^ package for crystal structure relaxations were used throughout this investigation. The N$_6$–N$_2$ discovery follows the same methodology we implemented to unravel the crystal structure of N$_5$AsF$_6$^51^. The N$_5$AsF$_6$ HEDM, which marked the history of polymeric nitrogen as the first synthesized polynitrogen after the discovery of N$_3^-$ back in the 1800's^52–54^, was highly explosive and impossible to characterize by XRD and spectroscopy techniques during data acquisition. The crystal structure of this pentanitrogen as C$_{2V}$ N$_5^+$ was only achieved by implementing GGA^55^ functionals within the PBEsol^56^ formalism. The importance of such a methodology lies in GGA PBEsol greater potential in the systematic lowering of the lattice constants compared to other GGA functionals. This improves equilibrium properties of densely packed solids such as PN and their surfaces^56^. On the other hand, starting with a crystal with N$_8$ stoichiometry will eventually lead to unwanted solids such as $\alpha$- and $\epsilon$-N$_2$ that are low pressure and low temperature phases. To remedy this situation, we took advantage of the molecular crystal approach of the algorithm to start from predefined blocks of N$_6$ and N$_2$. The rationale behind this choice is threefold: (1) N$_6$ in a C$_{2h}$ symmetry has been reported as a stable molecule in the gas phase^14^, (2) N$_{6}$ in this symmetry has the potential to be synthesized from two joining N$_3^-$, N$_3^*$ and N$_3^+$ and (3) The natural stability of N$_2$ will enhance the overall stability of the crystal. Moreover, we intentionally avoided to run the algorithm at zero pressure since, though we set the N$_6$ and N$_2$ initial blocks which should be maintained till reaching the end of the evolutionary search, the tendency to reach N$_2$ solids for nitrogen is overwhelming and would definitely undermine potential candidates throughout the search. We thus picked 5 GPa as the pressure for our runs. This is far away from the range where polymeirc nitrogen compounds such as cg-PN and molecular N$_8$ are known to form. Additional information about the USPEX^32–34^ methodology used here can be found in Supplementary Note 1. At 5 GPa, the best 20 energetically favorable structures are depicted in Fig. 2 where their positions along the evolutionary search are plotted against their volume (Å/atom) and enthalpy shift (meV/atom) from the best structure. Structures (a) and (b) of Fig. 2 are about 342 mev/atom apart from the third structure found and eventually bigger shift from the rest. This is a huge margin for a typical USPEX^32–34^ search and is a validation of the methodology applied to converge to the most favorable structure (See Supplementary Note 2 and Supplementary Table 2). More importantly, this stability gain acquired by the structures (a) and (b) is also reflected through their higher symmetry as they both adopt a monoclinic structure in the space group C$_{2h}(2/m)$ with comparable lattice constants and volume. Structure c) on the other hand exhibits a symmetry lowering to triclinic in the space group C$_{i}({\overline{1}})$ with a noticeable twist in the orientation of N$_2$ molecules with respect to N$_6$. The N$_6$-N$_2$ system thus found is characterized by parallel plans containing N$_6$ and N$_2$ molecules that alternate in a way that every N$_6$ molecule has 4 N$_2$ molecules as nearest neighbors and vice versa (See Supplementary Note 3 and Supplementary Fig. 1 for different views of this crystal arrangement). Upon lowering the pressure to zero and re-optimizing structure (a), the monoclinic symmetry is broken to triclinic with a significant angle twist of N$_2$ with respect to N$_6$. Up to now, this structure is proven in this investigation to be more favorable than the two predicted and synthesized cg-PN and 2N$_8$ polyintrogens as well as the molecular solid made of the C$_{2h}$ N$_6$ from Greschner et al^57^. In the 0-30 GPa range of Fig. 3, cg-PN becomes more favorable only after 20 GPa. With respect to 2N$_8$ and the N$_6$ solid, N$_6$–N$_2$ is energetically more stable throughout the whole pressure range covered by over 100 meV/atom up to 15 GPa, this margin shrinks slowly as the pressure goes up. Further comparison can be made with the most recently reported bipentazol molecular solid N$_{10}$ in the work of Bundarchuk^44^. Among the three phases reported (P$_{21}$ , I$_{222}$ and P$_{222}$ ), P$_{21}$ is more favorable than 2N$_8$ by about 80 mev/atom. This sets the structure we report here to be the most energetically favorable structure reported so far.

### Dynamical, mechanical and thermodynamical stability. Phonon and vibrational spectra

**Figure 4:**
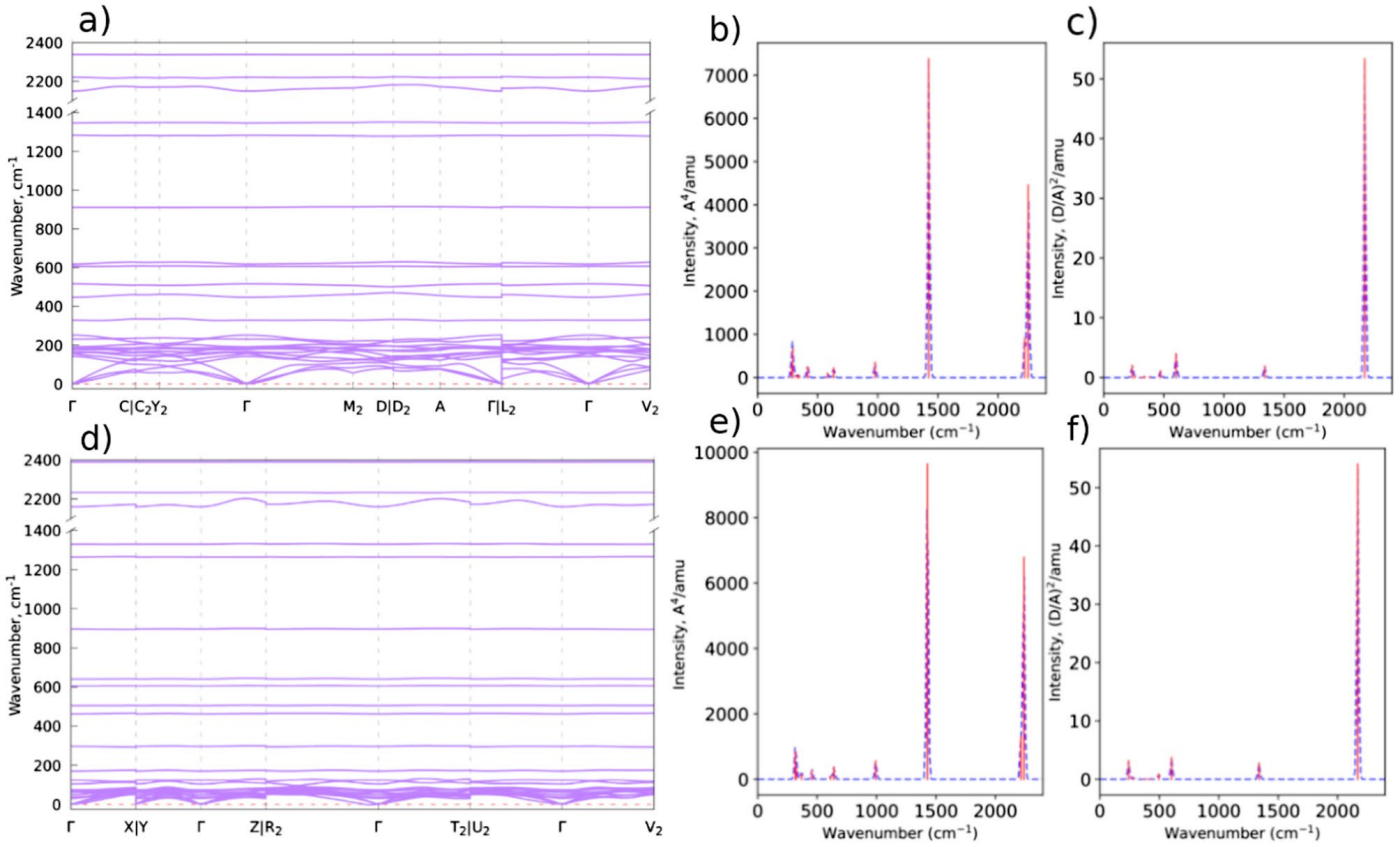
The N$_6$–N$_2$ system Calculated phonon dispersion along the high symmetry points of the first Brillouin zone, the predicted Raman and the IR spectra at 5 GPa [(**a**) , (**b**) and (**c**), respectively]. The same properties of N$_6$–N$_2$ at 0 GPa [(**d**), (**e**) and (**f**), respectively].

We adopted the notations and crystallographic directions suggested in reference^58^ for N$_6$–N$_2$ to obtain the phonon dispersion using the DFT perturbation theory^59^. At 5 GPa the monoclinic structure of point group C$_{2h}$(2/m) (see Supplementary Note 4 for the Crystallographic Information File (CIF)) follows the k-path: $\Gamma$-C|$C_2$-$Y_2$-$\Gamma$-$M_2$-D|$D_2$-A-$\Gamma$|L$_2$-$\Gamma$-V$_2$. Meanwhile, the triclinic structure of point group C$_{i}(\overline{1})$ at 0 GPa follows the k-path: $\Gamma$-X|Y-$\Gamma$-Z|R$_2$-$\Gamma$-T$_2$|U$_2$-$\Gamma$-V$_2$. Along the high symmetry points of the first Brillouin zone, the phonon dispersion at 5 GPa (Fig. 4-a)) and 0 GPa (Fig. 4-d) shows good dynamical stability as all modes are positive. The slopes of the acoustic branch at $\Gamma$ from any direction are almost identical which is an indication of the isotropic elasticity of this material. Moreover, both structures keep the same phonon features which suggests that the N$_6$-N$_2$ system has almost the same crystal structure. This is in spite of the slight symmetry breaking that takes place upon lowering pressure. This is further confirmed in the predicted Raman spectra (Fig. 4-b and -d. for 5 GPa and 0 GPa), respectively) as well as the IR activity (Figs. 4-c and -e. for 5 GPa and 0 GPa, respectively). The discrepancy between the normal modes upon varying pressure is in the 10–20 cm$^{-1}$ range and is a consequence of the bond length change with the pressure load. In addition, as revealed from the vibrational spectra of Fig. 4, this structure will be very distinguishable from other polymeric nitrogen compounds by its signature strong Raman signal at about 1425 cm$^{-1}$. In contrast, the mode in the vicinity of 2170 cm$^{-1}$ will be almost the only mode in the IR spectrum, this is a common vibrational frequency among many polynitrogens including all azide salts and the N$_8$ molecular crystal. Raman is thus the technique of choice to characterize this polynitrogen if a synthesis is proven to be successful in the future.

The (C$_{IJ}$) elastic constants of the N$_6$–N$_2$ system in its 5 GPa monoclimic phase and 0 GPa triclinic phase are provided in the Supplementary Note 5. We followed the stricter mechanical stability conditions for such low symmetry solids as discussed in the work of Mouhat et al.^60^ All the leading principal minors of the (C$_{IJ}$) tensor are positive for both phases and hence, the mechanical stability requirements are fully satisfied. However, distinct differences in the mechanical properties between the two phases are apparent as shown in Table 1. For instance, the small values of the elastic properties of the C$_{2h}(2/m)$ are comparable to those of ductile materials such as lead. These mechanical properties are further reduced for the C$_{i}({\overline{1}})$ phase, making the N$_6$-N$_2$ system a very soft material such as polymers.

**Table 1:** Mechanical constants (Bulk, Young and Shear moduli and Poisson ratio) of the N$_6$–N$_2$ polynitrogen at 5 and 0 GPa.

	B (GPa)	E (GPa)	G (GPa)	n
C$_{2h}$(2/m) , 5 GPa	21.52	26.94	10.43	0.29
C$_{i}({\overline{1}})$ , 0 GPa	1.05	2.68	1.86	0.04

**Figure 5:**
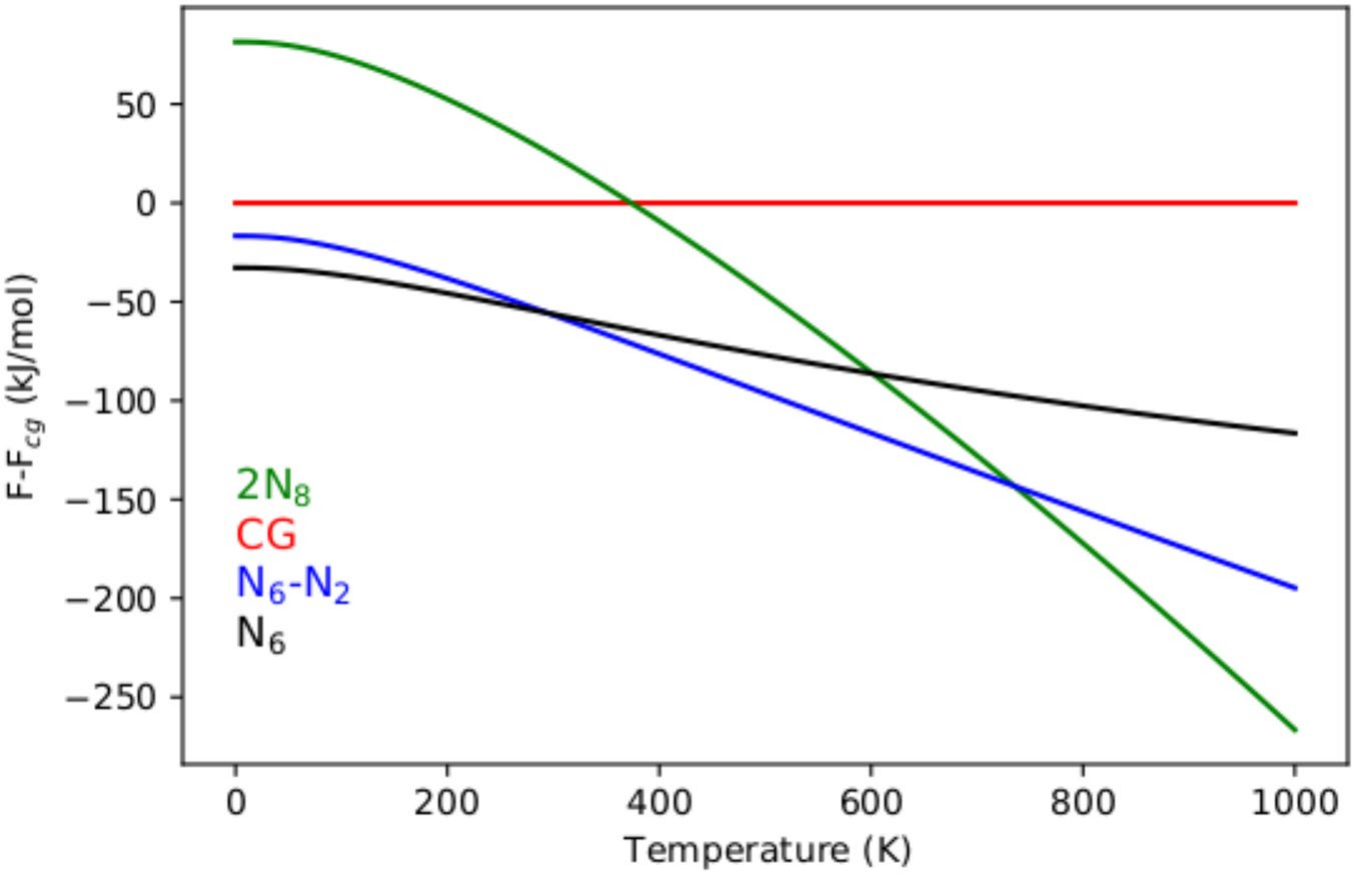
Zero-pressure Helmholtz free energy as a function of temperature for N$_6$–N$_2$, cg-PN, 2N$_8$ and N$_6$. cg-PN Helmholtz free energy is taken as a reference.

First-principal calculations based on density functional theory are often lacking finite temperature treatments. While this is generally acceptable for compounds with properties that are not much affected by temperature, a compound like cg-PN required a high temperature of 2000 K to be synthesized. To overcome the uncertainty surrounding stability of polymeric nitrogen compounds at finite temperature, we investigated the zero-pressure N$_6$–N$_2$ system under this investigation in comparison to the competing phases of cg-PN, 2N$_8$ and the N$_6$ solid. While cg-PN is regarded as a reference for its historical importance and 2N$_8$ for its significance as one of the few exotic polynitrogens that were successfully synthesized, the N$_6$ is chosen for its relevance to the system of this study as it also adopts the C$_{2h}$ symmetry. By limiting the calculations to P=0 GPa, the Helmholtz free energy F(T) offers much insight regarding polymeric nitrogen stability between the known phases. Figure 5 shows the results of the free energy as a function of temperature. The curves follow the same trend as tempretaure is increased up to 1000K. However, the N$_6$–N$_2$ system prevails as more favorable than 2N$_8$ , cg-PN and N$_6$ from room temperature and up to 750 K. Only at higher temperatures that the 2N$_8$ becomes more favorable. This explains the necessary laser heating to stabilize cg-PN at 2000K. At T=0 K , The Helmholtz free energy favors the N$_6$ system over N$_6$–N$_2$ which is in disagreement with the results of Fig. 3. This discrepancy is due to the zero-point energy contribution in the calculation of free energies which is absent in the ground state calculations within DFT under the Born-Oppenheimer approximation. With the phonopy package, the Helmholtz free energy is extracted from $F=\frac{1}{2} \sum _{qj}\hbar \omega _{qj} + K_{B}T \sum _{qj} \ln [1-exp(-\hbar \omega _{qj}/K_{B}T)]$ ($\omega _{qj}$ is the phonon mode at the {q,j} set, $\hbar$ the reduced Max Planck constant, K$_B$ the Boltzman constant and T is the temperature). The zero point energy that arises from the first term at T=0 K is only attributed to the summation over phonon frequencies in the first Briouillin zone. The N$_6$–N$_2$ system of our investigation is one N$_2$ molecule more per unit cell than N$_6$. This is responsible for increasing the zero point energy of N$_6$–N$_2$ and thus favoring N$_6$ at 0 K (See Table 2 for the results of the calculated vibrational zero-point energies of all the phases discussed). This aspect is actually persistent in the whole cryogenic regime up to ambient conditions. Also noticeable is that at high temperatures, the N$_6$–N$_2$ system becomes less favorable than the 2N$_8$ system only after 750 K.

**Table 2:** The calculated zero-point energies (ZPE) of N$_6$, N$_6$–N$_2$, CG and 2N$_8$.

Polynitrogen phase	N$_6$	N$_6$–N$_2$	CG	2N$_8$
ZPE (kJ/mole)	65.045	81.116	97.805	179.315

### NBO investigation and electronic structure

**Figure 6:**
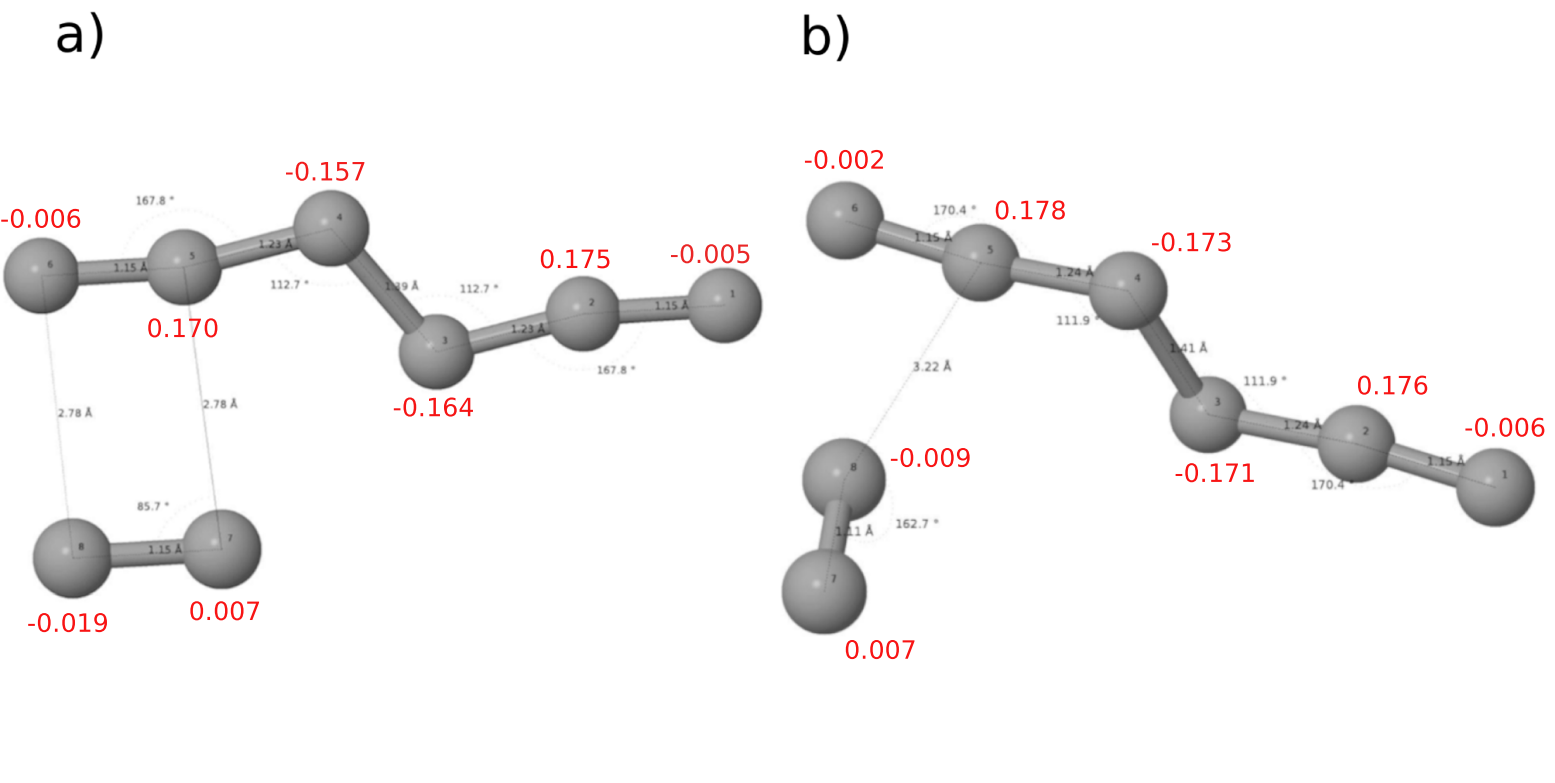
Intra- and inter-molecular bond lengths and angles along with the natural charge distribution from natural bonding orbital (NBO) analysis in the N$_6$–N$_2$ system: (**a**) for the C$_{2h}$(2/m) phase found at 5 GPa and (**b**) for the C$_{i}(\overline{1})$ found at 0 GPa.

As depicted in Fig. 6, the natural bonding orbital (NBO) analysis was used in this study to get a closer perspective into the intramolecular and intermolecular bonding that take place within the molecular crystal units. Structurally, the two different phases C$_{2h}(2/m)$ in Fig. 6-a and C$_{i}({\overline{1}})$ in Fig. 6-b are similar regarding bond lengths and angles, the slight variations within each fragment are only due to the pressure variation between the two phases. However, the difference seen in the orientation of the N$_2$ molecule with respect to N$_6$ which goes from 87.5$^{\circ }$ in the C$_{2h}(2/m)$ phase to 162.7$^{\circ }$ in the C$_{i}({\overline{1}})$ is huge. This is the main reason for the symmetry breaking between the two phases. In both structures, the terminal bonds in N$_6$ (N$_1$–N$_2$ and N$_5$–N$_6$ at 1.15 Å) have the characteristics of a triple bond. The N$_2$–N$_3$ and N$_4$–N$_5$ at 1.2 Å along with the central bond N$_3$–N$_4$ at 1.4 Å are single (More details on bonding is provided in Supplementary Note 6). The N$_2$ molecules within the crystal keep their natural triple bond at 1.11 Å. The separation between N$_6$ and N$_2$ which is indicative of the ionic bonding character in this crystal changes from 2.78 in the C$_{2h}(2/m)$ phase to 3.22 Å in the C$_{i}({\overline{1}})$. While the change in this separation in addition to N$_2$ orientation does not alter the natural charge distribution within N$_6$ dramatically, the end terminal of N$_2$ exhibits a charge depletion. The NBO investigation does not consider the boundary conditions of the overall crystal as only two neighboring N$_6$ and N$_2$ were considered for this calculation. The natural charge will be evenly distributed on both atoms of N$_2$ to keep its neutrality. The NBO serves here as only a qualitative approach to show the charge transfer that takes places between the molecular crystal constituents. In fact, N$_6$ takes a larger share from the overall natural charge to maintain its stability within the crystal. The lattice environment is thus believed important for providing the metastability required for this crystal to exist.

## Methods

Quantum ESPRESSO^49,50^, was used as the ab initio density functional theory (DFT^47,48^) engine to carry all the structural optimizations, the electronic structure, the phonon calculations, and the predicted IR/Raman spectroscopy data. To investigate the polymeric nitrogen compounds in this work at finite temperatures we used the finite displacement method as implemented in the phonopy^61^ package to extract the Helmholtz free energy as a function of temperature. USPEX^32–34^ (version 10.4) was used to conduct the ab initio evolutionary search for the system under investigation. We adopted the molecular crystal approach embedded in the software to start the search from predefined blocks of a N$_8$ system consisting of predefined molecular units in the form of one C$_{2h}$ N$_6$ and one N$_2$ units (See Supplementary Note 1 for more details). The algorithm was initiated with a pool of 30 randomly generated structures. The subsequent generations were produced with 50% heredity, 20% randomness and the remaining 30% equally generated by permutation, softmutation and lattice mutation. Each structure produced was structurally optimized by Quantum ESPRESSO^49,50^ in five steps starting with two successive atomic relaxations followed by three variable cell relaxations. This is done by enforcing stricter criteria on energy and forces on the atoms as the algorithm proceeds from step one to step five. The k-points resolution was also decreased after each step reaching its strictest value of 2$\pi$ Å$^{-1}$ with a kinetic energy cutoff of 680 ev. PBEsol^56^ GGA^55^ functionals were utilized for DFT^47,48^ calculations regarding all relaxation steps and phonon calculations. This follows our findings dealing with N$_5$AsF$_6$ crystal structure prediction^51^ where only GGA PBEsol was able to polymerize nitrogen as C$_{2V}$ N$_5^+$ within the crystal. The C$_{2h}$(2/m) phase found at 5 GPa and C$_{i}({\overline{1}})$ found at 0 GPa using PBEsol and was reoptimized by PBE^55^ and LDA^48^ to assess the impact of the level of theory on the lattice parameters (See Supplementary Table 1). van der Waals forces were incorporated in the calculation via dft-d3^62^ as implemented in Quantum Espresso. To take full advantage of density functional perturbation theory^59^ to predict the Raman/IR spectra, we switched to LDA^48^ functionals as third derivatives are not accessible with any GGA functional in the Quantum ESPRESSO^49,50^ package. This was done following a reoptimization of the structures of interest within LDA framework. The coordinates of the molecular building blocks of the N$_6$–N$_2$ system were utilized to build the structure for the GamessUS^63^ linked with nbo.6^64^ to investigate the inter/intra-molecular aspect of the system as well as the natural charge distribution and bonding characters within the molecules. For the structural visualization and producing the figures of this manuscript, Xcrysden^65^, VESTA^66^ and Jmol^67^ were used.

## Conclusions

Since its inception back in the 1980s, the field of polymeric nitrogen entered the era of materials discovery as a potential compound with immense applications for the energy sector and considerable environmental impact. This HEDM witnessed a plethora of discoveries of phases under different conditions, most of them are yet to be synthesized. If cg-PN is considered the pinnacle of these phases, a full-scale synthesis in our opinion is still complex to achieve with a substantial yield. In this investigation, we implemented the ab initio assisted evolutionary algorithm within USPEX methodology to discover the new polymeric nitrogen N$_6$–N$_2$ phase at relatively lower pressures. The system was proven dynamically and mechanically stable even at zero pressure. The readily available precursors in the form of N$_3^*$ for a potential synthesis gives this system a higher chance to be discovered. This work was made possible because of the powerful USPEX algorithm that culminated a lot of success since 2006 for materials discovery. For instance, discovering cg-PN as a potential PN required strong ingenuity. An evolutionary algorithm such as USPEX is capable of finding this high-pressure phase in record computational time and without any constraints imposed on the system. The N$_6$–N$_2$ system is believed to be easily distinguishable from other polymeric nitrogen compounds due to its high Raman activity at about 1425 cm$^{-1}$. The zero-pressure phase of this system is shown to stem from a symmetry breaking of the 5 GPa phase due to a change in the orientation of N$_2$ molecules within the crystal.

## Supplementary Information

Supplementary Information.

## Data Availability

Data underlying the results of this study are included in the published article and its supplementary information files. Further details may be obtained from the authors upon reasonable request.
