# Supplementary material for: A polymeric nitrogen N6–N2 system with enhanced stability at low pressure
Source: Sci Rep. 2022 Sep 12;12:15312. doi: 10.1038/s41598-022-19080-0 (PMC9468181; doi:10.1038/s41598-022-19080-0)
Supplement: Supplementary file 1 — Supplementary Information. [file 41598_2022_19080_MOESM1_ESM.pdf]

A polymeric nitrogen N_6-N_2 system with enhanced stability at low pressure

El Mostafa Benchafia[1], Xianqin Wang [2], Zafar Iqbal[3] & Sufian Abedrabbo[1,4]

Abstract

Postulated in 1992 and synthesized in 2004 above 2000 K and 110 GPa, the singly-bonded nitrogen cubic gauche crystal (cg-PN) is still considered to be the ultimate high energy density material (HEDM). The search however has continued for a method to synthesize cg-PN at more ambient conditions or find HEDMs which can be synthesized at lower pressure and temperature. Here, using ab initio evolutionary crystal prediction techniques, a simpler nitrogen-based molecular crystal consisting of N_6 and N_2 molecules is revealed to be a more favorable polynitrogen at lower pressures. The energetic gain of ~534 meV/atom over cg-PN and ~138 meV/atom over the N_8 molecular crystal at zero pressure makes the N_6-N_2 system more appealing. Dynamical and mechanical stabilities are investigated at 5 and 0 GPa, and vibrational frequencies are assessed for its Raman and IR spectra. The prospects of an experimental synthesis of the N_6-N_2 polymeric system compared to cg-PN is higher because the C_{2h} symmetry of N_6 within this crystal would to be easier to target from the readily available N_3^- azides and the observed N_3^+ and N_3^* radicals.

[1]Department of Physics, Khalifa University, Abu Dhabi, UAE. [2]Department of Chemical, Biological and Pharmaceutical Engineering, New Jersey Institute of Technology, Newark, NJ 07102, USA. [3]Department of Chemistry and Environmental Science, New Jersey Institute of Technology, Newark, NJ 07102, USA. [4]Department of Physics, The University of Jordan, Amman, Jordan. Correspondence and requests for materials should be addressed to Z.I (zafar.iqbal@njit.edu) and S.A (email: *sufian.abedrabbo@ku.ac.a*e).

Supplementary Note 1: The detailed USPEX search and Quantum Espresso parameters

The molecular crystal variant implemented in the USPEX[1–3] code is a powerful tool that is considered a constrained approach. With this methodology, the search is forced to look for the most favorable structure that contains the building molecular units of the crystal. Presented in a z-matrix formulation, the following N_6 and N_2 units were introduced to USPEX for the evolutionary algorithm search:

Molecular C_{2h} N_6

Number of atoms: 6

**N**	**0.0039**	**-0.1419**	**-2.7555**	**0**	**0**	**0**	**1**
**N**	**0.0004**	**0.0857**	**-1.6343**	**1**	**0**	**0**	**1**
**N**	**-0.0043**	**0.5276**	**-0.4758**	**2**	**1**	**0**	**1**
**N**	**-0.0043**	**-0.5276**	**0.4758**	**3**	**2**	**1**	**0**
**N**	**0.0004**	**-0.0857**	**1.6343**	**4**	**3**	**2**	**0**
**N**	**0.0039**	**0.1419**	**2.7555**	**5**	**4**	**3**	**0**

and

Molecular N_2

Number of atoms: 2

**N**	**0.0000**	**0.0000**	**-0.5511**	**0**	**0**	**0**	**1**
**N**	**0.0000**	**0.0000**	**0.5511**	**1**	**0**	**0**	**1**

A population of 30 randomly structures initiate the USPEX run as the first generation. The quantum espresso package[4,5] is then utilized to perform geometry optimization in 5 consecutive steps (two relaxations of atoms within the solids produced and three variable cell relaxations where the cells are also subjected to optimization). All consecutive generations follow a criterion that permits structures to be produced by 50% heredity, 20% randomness, 10% permutation, 10% softmutation and 10% lattice mutation. Apart from the molecular constraint imposed on the system, no other restrictions were imposed. Quantum Espresso optimizations were performed by the density functional theory (DFT [6,7]) with PBEsol[8] functionals which are found to be a reliable mechanism that is suitable for polymeric nitrogen (Our work[9] on N_5AsF_6 is an example of the success of PBEsol in successfully polymerizing nitrogen where other GGA[10] and LDA[11] functionals terribly failed). The k-points resolutions in each optimization step was set as $2\pi\times 0.16Å^{-1}$ in the first step, $2\pi\times 0.14Å^{-1}$ in the second, $2\pi\times 0.12Å^{-1}$ in the third, $2\pi\times 0.10Å^{-1}$ in the fourth and $2\pi\times 0.08Å^{-1}$ in the fifth step. The kinetic energy cuttoffs were at 680 ev in all DFT optimization steps at the set value of 5 GPa for pressure. Convergence threshold for self-consistency, the total energy and the forces on atoms are stricter after each step, reaching values of 10^{-8}, 10^{-5} ev/atom and 10^{-3} ev$Å^{-1}$, respectively.

Supplementary Note 2: Illustration of some of the best structures found

The best structures found in the USPEX search led to two structures with distinctive enthalpy margin than the rest (crystallographic data file (CIF) shown below). The first two structures are both monoclinic in the C_{2h}(2/m) symmetry group. An illustration of this finding is depicted in Supplementary Table 1 where the best five structures are shown.

***Supplementary* Table 1:** The 5 best structures found. Structures 691 and 708 are of the same symmetry group, comparable volumes with a minuscule enthalpy difference.

Structure ID	Enthalpy (ev/atom)	Volume (Å^3/atom)	Symmetry
691	-272.6204	11.4893	12
708	-272.6203	11.4858	12
1197	-272.2784	11.3865	2
1166	-272.2781	11.3908	2
1217	-272.2781	11.3908	2

Supplementary Note 3: Different projected views of the N_6-N_2 crystals are presented in ***Supplementary* Figure 1**. In the 5 GPa monoclinic C_{2h}(2/m) phase (top), the crystal constituents made of N_6 and N_2 molecular units are contained in parallel plans. N_2 molecules axes are almost parallel to the axis of N_6 molecule. Upon lowering pressure to 0 GPa, the monoclinic phase also lowers its symmetry to a triclinic $C_i(\bar{1})$ phase. In this phase, the crystal arrangement is distorted and the planes containing N_2 molecules are penetrating the planes containing N_6.

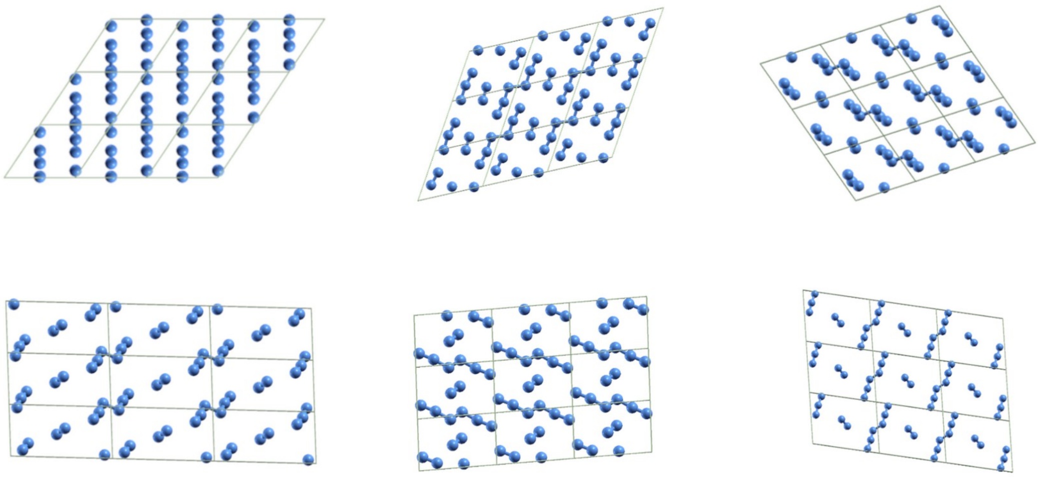

Supplementary Figure. 1: Comparison of different views of the N_6-N_2 crystals in (top) the 5 GPa monoclinic phase C_{2h}(2/m) and (bottom) the 0 GPa $C_i(\bar{1})$ triclinic phase.

Supplementary Note 4: crystallographic data file (CIF) of the best two structures.

```
data_findsym-STRUC-691
_chemical_name_common                  'findsym-STRUC-691'
_cell_length_a                         8.430840
_cell_length_b                         4.828000
_cell_length_c                         5.309000
_cell_angle_alpha                      90.000000
_cell_angle_beta                       58.309769
_cell_angle_gamma                      90.000000
_cell_volume                           183.877967
_space_group_name_H-M_alt              'C 2/m'
_space_group_IT_number                 12

loop_
_space_group_symop_operation_xyz
   'x, y, z'
   '-x, -y, -z'
   '-x, y, -z'
   'x, -y, z'
   'x+1/2, y+1/2, z'
   '-x+1/2, -y+1/2, -z'
   '-x+1/2, y+1/2, -z'
   'x+1/2, -y+1/2, z'

loop_
   _atom_site_label
   _atom_site_occupancy
   _atom_site_fract_x
   _atom_site_fract_y
   _atom_site_fract_z
   _atom_site_adp_type
   _atom_site_U_iso_or_equiv
   _atom_site_type_symbol
   N        1.0    -0.243100    0.000000    0.174800    Uiso  ? N
   N        1.0    -0.362200    0.000000    0.130000    Uiso  ? N
   N        1.0     0.488400    0.000000    0.139900    Uiso  ? N
   N        1.0     0.069000    0.000000    0.489800    Uiso  ? N
```

Supplementary Note 5: Using the thermo_pw/QUANTUM ESPRESSO[4,5] code, the elastic constants can be extracted. The method works by applying a given strain and calculating the stress. The 6 by 6 elastic tensor (three tensile and three shear components) is then computed and all other mechanical properties derived. The elastic tensors of the N_6-N_2 systems in both symmetries are:

5 GPa monoclinic phase:

$$C_{IJ}(kbar)=\begin{pmatrix} 320.93598 & 149.98633 & 163.05659 & 0.00000 & -19.05544 & 0.00000 \\ 156.86040 & 381.95974 & 107.32088 & 0.00000 & -26.69223 & 0.00000 \\ 161.69504 & 166.82327 & 543.95514 & 0.00000 & 41.38968 & 0.00000 \\ 0.00000 & 0.00000 & 0.00000 & 82.01879 & 0.00000 & -5.35133 \\ -15.07886 & -29.41750 & 19.60471 & 0.00000 & 79.23346 & 0.00000 \\ 0.00000 & 0.00000 & 0.00000 & -5.71412 & 0.00000 & 116.45295 \end{pmatrix}$$

0 GPa triclinic phase:

$$C_{IJ}(kbar)=\begin{pmatrix} 25.58617 & 1.69821 & 7.41963 & 0.05918 & 0.02979 & -0.65231 \\ 0.81794 & 31.54236 & -5.12217 & -3.33603 & -2.10106 & 31.23620 \\ 6.53150 & 10.21108 & 21.08444 & 1.82008 & -3.06189 & 3.79252 \\ 4.62266 & -6.09979 & 15.10504 & 15.33919 & 0.01210 & -20.94627 \\ 0.37604 & -1.24202 & -0.65671 & -1.75293 & 11.53216 & -9.26625 \\ -6.09973 & 2.45832 & -11.77613 & -4.39838 & -5.74193 & 27.59046 \end{pmatrix}$$

Supplementary Note 6: Distinguishing the nature of the bonds involved in the N_6-N_2 system in both symmetries is not only derived from the bond length. Oftentimes, bond lengths are a good indication of the kinds of bonds involved: with nitrogen entities, ~1.1 Å is characteristic of a triple bond, ~ 1.2 Å for double and ~1.4 Å for single bonds. However, the true character of any chemical bond is directly derived for the natural bonding orbital theory (NBO) by looking at the Lewis character. Extracts of this investigation is given below for the N_6-N_2 system in both symmetries.

5 GPa monoclinic phase:

.

.

17. (0.99897) BD (1) **N1- N2**

(42.95%) 0.6554* N1 s(28.16%)p 2.54(71.52%)d 0.01(0.32%)

(57.05%) 0.7553* N2 s(49.79%)p 1.01(50.14%)d 0.00(0.07%)

18. (0.99698) BD (2) **N1- N2**

(48.57%) 0.6969* N1 s(0.00%)p 1.00(99.60%)d 0.00(0.39%)

(51.43%) 0.7171* N2 s(0.00%)p 1.00(99.83%)d 0.00(0.17%)

19. (0.98747) BD (3) **N1- N2**

(40.69%) 0.6379* N1 s(1.72%)p56.95(97.84%)d 0.26(0.44%)

(59.31%) 0.7701* N2 s(1.11%)p88.71(98.79%)d 0.09(0.10%)

20. (0.99529) BD (1) **N2- N3**

(55.33%) 0.7439* N2 s(48.96%)p 1.04(50.98%)d 0.00(0.06%)

(44.67%) 0.6683* N3 s(27.19%)p 2.67(72.58%)d 0.01(0.23%)

21. (0.95720) BD (1) **N3- N4**

(49.92%) 0.7066* N3 s(20.02%)p 3.99(79.79%)d 0.01(0.19%)

(50.08%) 0.7077* N4 s(20.03%)p 3.98(79.78%)d 0.01(0.19%)

22. (0.99524) BD (1) **N4-N5**

(44.62%) 0.6680* N4 s(27.25%)p 2.66(72.53%)d 0.01(0.22%)

(55.38%) 0.7442* N5 s(48.92%)p 1.04(51.02%)d 0.00(0.07%)

23. (0.99869) BD (1) **N5- N6**

(57.03%) 0.7552* N5 s(49.69%)p 1.01(50.23%)d 0.00(0.07%)

(42.97%) 0.6555* N6 s(28.17%)p 2.54(71.51%)d 0.01(0.32%)

24. (0.99577) BD (2) **N5- N6**

(51.51%) 0.7177* N5 s(0.04%)p99.99(99.79%)d 4.07(0.17%)

(48.49%) 0.6963* N6 s(0.01%)p99.99(99.60%)d36.17(0.39%)

25. (0.98750) BD (3) **N5- N6**

(59.26%) 0.7698* N5 s(1.21%)p81.30(98.69%)d 0.08(0.10%)

(40.74%) 0.6383* N6 s(1.81%)p53.91(97.75%)d 0.24(0.44%)

26. (0.99998) BD (1) **N7- N8**

(50.16%) 0.7082* N7 s(0.21%)p99.99(99.39%)d 1.89(0.40%)

(49.84%) 0.7060* N8 s(0.18%)p99.99(99.42%)d 2.24(0.40%)

27. (0.99968) BD (2) **N7- N8**

(50.45%) 0.7103* N7 s(1.73%)p56.45(97.87%)d 0.23(0.39%)

(49.55%) 0.7039* N8 s(1.60%)p61.17(98.00%)d 0.25(0.40%)

28. (0.99915) BD (3) **N7- N8**

(50.06%) 0.7076* N7 s(32.68%)p 2.05(66.97%)d 0.01(0.35%)

(49.94%) 0.7067* N8 s(32.72%)p 2.05(66.93%)d 0.01(0.35%)

.

.

..

0 GPa triclinic phase:

.

.

17. (0.99902) BD (1) **N1- N2**
(43.14%) 0.6568* N1 s(28.65%)p 2.48(71.03%)d 0.01(0.32%)

(56.86%) 0.7541* N2 s(49.70%)p 1.01(50.23%)d 0.00(0.07%)

18. (0.99734) BD (2) **N1- N2**
(48.58%) 0.6970* N1 s(0.03%)p99.99(99.58%)d15.58(0.40%)

(51.42%) 0.7171* N2 s(0.04%)p99.99(99.79%)d 4.42(0.17%)

19. (0.98731) BD (3) **N1- N2**
(40.68%) 0.6378* N1 s(1.33%)p74.13(98.23%)d 0.33(0.44%)

(59.32%) 0.7702* N2 s(1.14%)p86.47(98.76%)d 0.08(0.09%)

20. (0.99558) BD (1) **N2- N3**
(55.54%) 0.7452* N2 s(48.99%)p 1.04(50.95%)d 0.00(0.06%)

(44.46%) 0.6668* N3 s(27.00%)p 2.69(72.76%)d 0.01(0.23%)

21. (0.95334) BD (1) **N3- N4**
(50.03%) 0.7073* N3 s(19.04%)p 4.24(80.77%)d 0.01(0.19%)

(49.97%) 0.7069* N4 s(18.99%)p 4.26(80.82%)d 0.01(0.19%)

22. (0.99555) BD (1) **N4- N5**
(44.49%) 0.6670* N4 s(27.06%)p 2.69(72.71%)d 0.01(0.23%)

(55.51%) 0.7451* N5 s(48.98%)p 1.04(50.96%)d 0.00(0.06%)

23. (0.99898) BD (1) **N5- N6**
(56.87%) 0.7541* N5 s(49.70%)p 1.01(50.23%)d 0.00(0.07%)

(43.13%) 0.6568* N6 s(28.65%)p 2.48(71.04%)d 0.01(0.32%)

24. (0.99717) BD (2) **N5- N6**

(51.44%) 0.7172* N5 s(0.03%)p99.99(99.80%)d 5.83(0.17%)

(48.56%) 0.6968* N6 s(0.02%)p99.99(99.58%)d17.91(0.40%)

25. (0.98719) BD (3) **N5- N6**

(59.32%) 0.7702* N5 s(1.16%)p85.23(98.75%)d 0.08(0.09%)

(40.68%) 0.6378* N6 s(1.35%)p72.74(98.21%)d 0.33(0.44%)

26. (1.00000) BD (1) **N7- N8**

(49.82%) 0.7059* N7 s(0.72%)p99.99(98.85%)d 0.61(0.43%)

(50.18%) 0.7083* N8 s(0.71%)p99.99(98.87%)d 0.60(0.43%)

27. (0.99999) BD (2) **N7- N8**

(49.86%) 0.7061* N7 s(1.69%)p57.95(97.88%)d 0.25(0.43%)

(50.14%) 0.7081* N8 s(1.69%)p57.79(97.88%)d 0.25(0.43%)

28. (0.99995) BD (3) **N7- N8**

(49.90%) 0.7064* N7 s(34.67%)p 1.87(64.97%)d 0.01(0.36%)

(50.10%) 0.7078* N8 s(34.97%)p 1.85(64.67%)d 0.01(0.36%)

.

.

.

***Supplementary Table* 2:** Lattice parameters of the N_6-N_2 polynitrogen at 5 and 0 GPa at

different levels of theory (PBEsol, PBE and LDA).

$C_{2h}(2/m)$, 5 GPa							
	a (Å)	b(Å)	c (Å)	α°	β°	γ°	Volume (Å³/atom)
PBEsol	8.43084	4.82800	5.30900	90.0000	58.30977	90.000	11.50569875
PBE	9.57240	4.90357	7.29560	90.0000	56.42900	90.000	11.83541875
LDA	8.6456	4.4459	6.6474	90.0000	54.66100	90.000	9.23680625
$C_i(\bar{1})$, 0 GPa							
	a (Å)	b(Å)	c (Å)	α°	β°	γ°	
PBEsol	3.73386	7.50883	5.71767	87.296	87.880	101.703	18.2539525
PBEsol DFT-D3(vdW)	3.46100	7.31900	5.07700	77.18100	91.17000	84.76300	15.5956
PBE	3.6967	7.3552	5.6185	88.351	88.141	102.296	18.63589
LDA	3.1219	6.4820	4.9567	90.503	92.076	101.623	12.27126

1 Lyakhov, A. O., Oganov, A. R., Stokes, H. T. & Zhu, Q. New developments in evolutionary structure prediction algorithm USPEX. *Computer Physics Communications* **184**, 1172-1182, doi:https://doi.org/10.1016/j.cpc.2012.12.009 (2013).

2 Oganov, A. & Glass, C. Crystal structure prediction using ab initio evolutionary techniques: Principles and applications. *The Journal of chemical physics* **124**, 244704, doi:10.1063/1.2210932 (2006).

3 Oganov, A., Lyakhov, A. & Valle, M. How Evolutionary Crystal Structure Prediction Works—and Why. *Accounts of chemical research* **44**, 227-237, doi:10.1021/ar1001318 (2011).

4 Giannozzi, P. *et al.* QUANTUM ESPRESSO: a modular and open-source software project for quantum simulations of materials. *Journal of Physics: Condensed Matter* **21**, 395502, doi:10.1088/0953-8984/21/39/395502 (2009).

5 Giannozzi, P. *et al.* Advanced capabilities for materials modelling with Quantum ESPRESSO. *Journal of Physics: Condensed Matter* **29**, 465901, doi:10.1088/1361-648x/aa8f79 (2017).

6 Hohenberg, P. & Kohn, W. INHOMOGENEOUS ELECTRON GAS. **Vol: 136**, doi:10.1103/PhysRev.136.B864. Physical Review (U.S.) Superseded in part by Phys. Rev. A, Phys. Rev. B: Solid State, Phys. Rev. C, and Phys. Rev. D; Journal Volume: Vol: 136; Other Information: Orig. Receipt Date: 31-DEC-65 (1964).

7 Kohn, W. & Sham, L. J. Self-Consistent Equations Including Exchange and Correlation Effects. *Physical Review* **140**, 1133-1142 (1965).

8 Perdew, J. P. *et al.* Restoring the Density-Gradient Expansion for Exchange in Solids and Surfaces. *Physical Review Letters* **100**, 136406, doi:10.1103/PhysRevLett.100.136406 (2008).

9 Benchafia, E. M., Wang, X., Iqbal, Z. & Abedrabbo, S. Predicting the crystal structure of N_5AsF_6 high energy density material using ab initio evolutionary algorithms. *Scientific Reports* **11**, 7874, doi:10.1038/s41598-021-86855-2 (2021).

10 Perdew, J. P. Density-functional approximation for the correlation energy of the inhomogeneous electron gas. *Physical review. B, Condensed matter* **33**, 8822-8824, doi:10.1103/physrevb.33.8822 (1986).

11 Kohn, W. & Sham, L. J. Self-Consistent Equations Including Exchange and Correlation Effects. *Physical Review* 140, 1133-1142 (1965).
